# Synthetic Electrospun Fiber Matrix in the Management of Acute Wounds Following Excision of Hidradenitis Suppurativa Lesions: A Prospective Pilot Study

**DOI:** 10.3390/polym17192563

**Published:** 2025-09-23

**Authors:** Michael Madueke, Frank Lau

**Affiliations:** 1 Tulane University School of Medicine, 1430 Tulane Ave, New Orleans, LA 70112, USA; umadueke@tulane.edu; 2 Keliomics, Inc., 4640 S. Macadam Ave, Portland, OR 97239, USA; 3 Louisiana State University Health Sciences Center, Department of Surgery, Section of Plastic & Reconstructive Surgery, 1542 Tulane Ave, New Orleans, LA 70112, USA

**Keywords:** tissue regeneration, hidradenitis suppurativa, surgical wound, synthetic matrix, synthetic electrospun fiber matrix, skin grafting, infection, dermal regeneration, electrospinning

## Abstract

Hurley Stage II or III hidradenitis suppurativa (HS) may necessitate surgical excision of diseased skin and subcutaneous fat for symptom control and disease management. These excisions result in open wounds in topographically challenging regions and typically cannot be primarily closed. This study evaluates the use of a synthetic electrospun fiber matrix (SEFM) as a post-resection regeneration template to accelerate re-granulation and improve subsequent skin graft incorporation. This prospective pilot study enrolled Hurley Stage II or III patients undergoing surgical resection of HS lesions. SEFM was applied to the resulting wounds in conjunction with negative pressure wound therapy (NPWT). Patients were monitored post-operatively for granulation tissue formation and underwent skin grafting once granulation was sufficient. Skin graft incorporation was assessed at follow-up visits. Complications, including graft loss (partial or complete) and infection, were assessed at each encounter. A total of 21 wounds in eight patients met the inclusion criteria and were enrolled. The average time to skin grafting was 14 ± 3.2 days. After grafting, the average graft incorporation was 71 ± 28%. No complications occurred during the study. These initial results indicate that by supporting granulation tissue formation, combined use of SEFM and NPWT may aid in successful engraftment of topographically challenging areas post-HS excision.

## 1. Introduction

Hidradenitis suppurativa (HS) is a progressive disease with no pharmacologic cure. It starts with chronic inflammation of pilosebaceous units, which leads to the formation of subcutaneous nodules [1,2]. These nodules progressively become larger and form painful abscesses (Hurley Stage I) [1,2]. As the disease progresses, the abscesses spontaneously drain via sinus tracts that erupt through the skin (Hurley Stage II) [3]. The perineal, perianal, and inguinal regions are most commonly affected, causing pain and impairing activities of daily living including ambulation, exercise, bowel movements, and sexual function [3]. The diseased areas progressively enlarge and become interconnected through multiple sinus tracts, forming permanent areas of infected, scarred tissues that cannot be resolved without surgical excision (Hurley Stage III) [4].

Wide excision of HS lesions is indicated when there is risk of fibrosis or architectural loss [5]. This surgical approach involves removal of lesions in addition to surrounding disease-free tissue such as subcutaneous fat or intertriginous skin [5]. Post-excision wounds can measure hundreds of square centimeters and often occur in intertriginous locations in which apocrine glands are most abundant [5,6]. A retrospective review encompassing 122 patients treated over 35 years reported an average post-excision wound size of 98 cm^2^ for males and 55 cm^2^ for females [7]. These post-excision wound sizes were similar to those reported in a separate retrospective review of 79 patients with 220 total operative sites, which reported a mean excised area of 72 cm^2^ [8]. The most excised areas reported are the axilla (45%), groin (20%), and buttocks, perineum, or scrotum (16%). Excision size has been reported to be greater in patients who have not experienced disease recurrence; however, it introduces new risks in terms of increased morbidity and a need for more complex approaches to wound closure [8].

Management options include primary closure, healing by secondary intention, split-thickness skin grafts (STSGs), and locoregional flaps [9]. Primary closure permits these surgical wounds to heal faster and with less contracture but is only indicated in smaller wound sizes [5]. Locoregional flaps may also be considered in reconstructing HS excision wounds, but these reconstructions can be complex and typically only utilized in instances of deep wounds in which vascular channels or nerves may be exposed [5]. Adipocutaneous flaps have demonstrated high rates of post-operative complications, including wound dehiscence, post-operative bleeding, and hematoma [10].

Secondary intention healing and healing with STSGs have reported similar recurrence rates (37.6% vs. 33%, respectively) in addition to positive cosmetic outcomes [5]. Wound closure with STSGs is typically faster than that of secondary intention healing, which may limit the risk associated with secondary infection of an open wound, especially in consideration of groin or buttocks locations [5]. While patients generally preferred secondary intention healing, satisfaction with STSG closure and increased mobility was increased with use of negative pressure wound therapy (NPWT) [5]. STSGs also lack the presence of hair follicles and sweat glands, which may be beneficial considering their role in the pathogenesis of HS [5]. Considering the pros and cons of these options, the approach to wound closure should be catered to each patient.

STSG following the development of a healthy granulation bed is the most common approach [6]. STSG reduces scar contracture, thereby preserving range of motion in the axillae and genital regions [11]. Accelerating the formation of a healthy granulation bed is imperative as it reduces wound care-related pain [12]. Dermal regeneration templates (DRTs) have historically been utilized to stimulate granulation tissue formation in an effort to quicken time to STSG application [13]. DRTs are designed to encourage fibroblast migration and neovascularization within the wound bed [13,14,15,16]. Once fully granulated and vascularized, the wound bed is maximized to support STSG integration [13,14,15,16]. DRTs are often either biologic or synthetic in their composition and typically consist of an overlying silicone layer designed to provide a biological barrier to protect from external contamination as well as to maintain moisture within the wound [13,14,15,16]. In the context of HS resection wounds, which often occur in areas with a high risk of contamination such as the perianal, perineal, and axillary regions, it is critical to ensure that the DRT is not at risk of becoming infected. Biologically based DRTs may be at a higher risk of developing infection, as the biologic components can encourage microbial growth [16]. One systematic review of a bilayer bovine collagen matrix identified an overall infection rate of 16.9% [17].

A synthetic wound healing matrix may offer a more ideal solution in the staging of post-HS excision wounds. A synthetic electrospun fiber matrix (SEFM) has been utilized to stage both surgical and traumatic wounds to STSG with minimal instances of infection and has encouraged granulation tissue formation in the presence of contamination within the wound bed [18,19,20]. SEFM is composed of two resorbable, synthetic polymers which are electrospun to mimic that of human extracellular matrix (ECM) [21,22]. The matrix contains both micron-scale and sub-micron fibers (i.e., hybrid-scale) and a porous structure which encourages fibroblast migration, proliferation, and subsequently revascularization. SEFM resorbs through hydrolysis over a period of 1–3 weeks, allowing for controlled offloading from the matrix to the newly formed tissue [21,22]. Prior retrospective studies of SEFM in chronic lower extremity wounds have reported an 85% closure rate within 12 weeks with no instances of infection and minimal inflammatory response [23]. These results were validated in a randomized controlled trial evaluating the healing rate of patients treated with SEFM vs. standard-of-care dressings in diabetic foot ulcers, in which SEFM demonstrated statistical superiority over standard-of-care dressings [24]. Various case reports evaluating SEFM use in both acute and colonized wounds have demonstrated robust granulation tissue formation, persistence upon exposure to bile, and decrease in wound exudate following SEFM application in conjunction with standard wound care [18,19,20].

Given these properties, we hypothesized that SEFM would accelerate granulation tissue formation and decrease the time between HS excision and skin grafting. In the present prospective pilot study, a synthetic electrospun fiber matrix (SEFM) was applied in conjunction with NPWT to post-excisional wounds in Hurley Stage II and III patients, as patients with these disease stages may be eligible for surgical excision based on the presence of sinus tracts [25]. Our primary objective was to quantify the time from HS excision and application of SEFM to the time of skin grafting. Secondary objectives included rates of skin graft incorporation and incidence of healing complications, including infection, bleeding, and hematoma formation.

## 2. Materials and Methods

This prospective, observational pilot study was conducted at a single academic medical center (Louisiana State University Health Sciences Center, New Orleans). The study was conducted in accordance with Good Clinical Practice guidelines and the Declaration of Helsinki, as well as other local regulatory laws and requirements. Local institutional approval was granted by the Institutional Review Board of Louisiana State University Health Science Center New Orleans (Protocol #4862, approved 13 January 2023). Patients older than 18, with grade II or III HS, and who consented for surgical removal and skin grafting were selected for this study. Patients with perianal or perineal wounds were required to undergo temporary diverting colostomy prior to resection to reduce the risk of infection secondary to fecal contamination of the wound bed and subsequent STSG. Exclusion criteria included active smokers, patients with a body mass index (BMI) of >45, and those with active autoimmune diseases. Vulnerable populations, such as incarcerated, unable to consent, or pregnant patients, were also excluded. All subjects provided written informed consent.

Radical excision of HS was performed in the operating room (OR) utilizing a tumescent-based approach by a single surgeon. In this technique, active HS lesions were excised with 2 cm margins using #22 blades after infiltrating tumescent solution (1 L of Ringer’s lactate with 1 ampule of epinephrine and 40 mL of 1% lidocaine) into subcutaneous layers. The deep margin of excision consisted of the hydro-dissected, relatively avascular layer deep to diseased tissue. Following excision and hemostasis, meshed SEFM (Restrata^®^, Acera Surgical, Inc., St. Louis, MO, USA) was applied to the wound bed and secured with staples. Negative pressure wound therapy (NPWT) was applied over the SEFM, with an interpositional sheet of fine mesh gauze occlusive dressing impregnated with petrolatum and 3% bismuth tribromophenate (Xeroform^®^) to prevent adherence of the NPWT sponge to the SEFM. NPWT changes and wound checks were performed weekly by the surgeon to assess granulation tissue formation and monitor for complications. After the wound was sufficiently re-granulated, determined by the surgeon’s assessment, the patient returned to the OR for application of an STSG. The STSG was harvested, meshed 1.5:1, secured to the granulation bed with staples, and bolstered with NPWT.

The primary outcome measure was time from SEFM placement to >90% wound bed granulation, as assessed weekly by the treating surgeon and documented by photographs. Secondary outcome measures included STSG engraftment rate and incidence of wound complications. STSG engraftment was measured by the percent of total wound area covered by the STSG at seven days post-operatively and reported as a continuous variable. Complications including infection, major bleeding, and hematoma formation were monitored throughout the entirety of the treatment course, from HS excision until final clinic visit. Wound monitoring and assessment of overall patient health were performed through institutional standard-of-care evaluation. Wound monitoring through evaluation of amount and type of exudate, peri-wound erythema, and edema were performed by the clinician at each encounter. Overall patient clinical status was assessed at each encounter through evaluation of vital signs, clinician assessment, and institutional standard-of-care pre-operative blood work. The presence of wound healing complications was determined by clinician evaluation. Infection was defined as any soft tissue infection that occurred at the excision site or immediate surrounding tissues. Major bleeding was defined as bleeding that required procedural intervention. Hematoma was defined as a subcutaneous collection of blood occurring within 48 h of surgery.

Data were collected prospectively at standard-of-care post-operative visit intervals and stored in a Health Insurance Portability and Accountability Act (HIPAA)-compliant, firewalled, access-controlled online database. Digital photographs of the wounds were obtained at each follow-up visit to document wound size and the presence of granulation tissue. Images were obtained at all post-STSG follow-up visits to document the percentage of graft incorporation. Any adverse events were assessed for and documented if observed. Demographic data including age, race, gender, and BMI were collected and reported using descriptive statistics. The primary outcome measure of time from SEFM to >90% wound bed granulation was assessed in each wound and documented as number of days from SEFM application to wound bed granulation. This was reported using the mean and standard deviation of all wounds treated in the study. Secondary outcomes included STSG engraftment rate and complications including infection, bleeding, and hematoma. Rate of STSG success was assessed in each wound and reported as the percent of the graft that successfully incorporated into the wound bed. Successful STSG engraftment was reported as the mean and standard deviation of all wounds grafted in the study, with instances of complete graft loss reported as 0% success. Complications were reported as a percentage of total wounds.

## 3. Results

### 3.1. Patient Demographics

A total of 21 wounds from eight patients met the inclusion criteria. All (100%) were enrolled in the study. Data from all 21 wounds were included in the analysis. The post-excision wound size averaged 174.8 ± 139.6 cm^2^. A majority of the excisions occurred in the axillary, vulvar, inguinal, and perianal regions. A breakdown of patient demographics and wound characteristics is available in Table 1.

### 3.2. Clinical Outcomes

**Figure 1:**
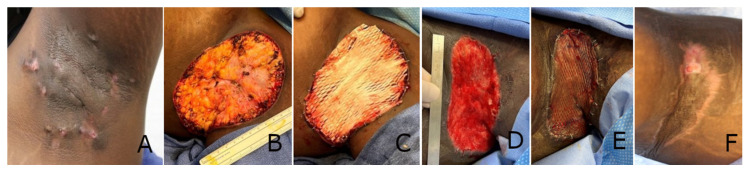
Progressive healing of a hidradenitis suppurativa (HS) excision wound. (**A**) Pre-operative appearance of nodules. (**B**) HS excision down to fat layer, resulting in a wound size of 14 cm × 10 cm. (**C**) Application of a meshed synthetic electrospun fiber matrix (SEFM) immediately post-resection. (**D**) The fully granulated wound bed demonstrating a 35% reduction in wound surface area 16 days after the index procedure. (**E**) A meshed split-thickness skin graft (STSG) applied to the wound bed at day 16. (**F**) Six weeks post-skin grafting, with a 98% graft incorporation observed.

**Figure 2:**
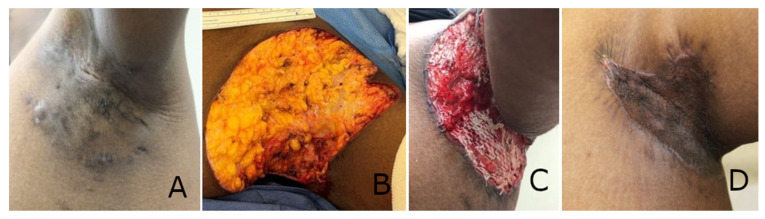
Progressive healing of an axillary hidradenitis suppurativa (HS) excision wound. (**A**) Pre-operative appearance of HS lesions. (**B**) HS excision down to fat tissue, with a resulting wound surface area of 21.5 cm × 18 cm. (**C**) The synthetic electrospun fiber matrix (SEFM) resorbing into the surgical wound bed with significant granulation tissue formation roughly 1 week after the index procedure. (**D**) The patient 8 weeks after skin grafting, with 100% incorporation and healing.

Progressive healing of all excision wounds was observed throughout the treatment period, as can be observed in Figure 1 and Figure 2.

**Figure 3:**
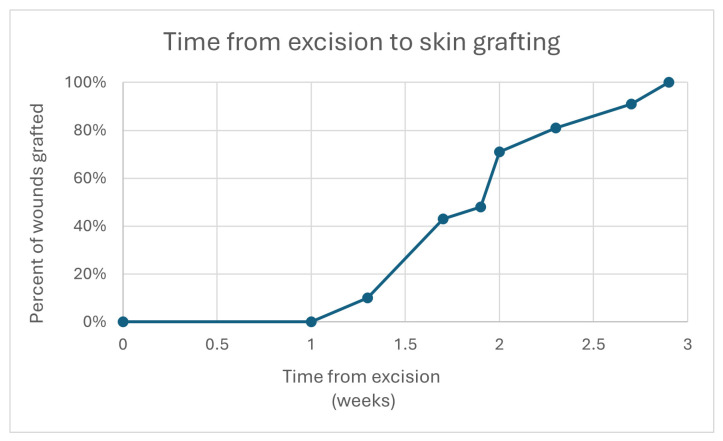
Time in weeks from hidradenitis suppurativa excision with synthetic electrospun fiber matrix application (SEFM) to skin grafting. Each wound is represented individually.

The mean time from excision surgery and SEFM application to STSG was 14 ± 3.2 days. All excisions resulted in full thickness wounds and extended into the subcutaneous adipose tissue layer. A progression of the time from initial excision to skin graft application can be observed in Figure 3.

Following STSG application, we assessed STSG incorporation weekly. The average graft incorporation at follow-up was 71% ± 28% (Table 2). The highest rates of graft incorporation were observed in axillary regions (95%). Lower rates of graft incorporation were observed in inguinal (63%) and perianal (40%) locations, likely due to greater contamination in those areas and challenges with shear forces during ambulation and standing. Two complete graft losses occurred, one in a perianal wound and another in the posterior scalp wound. The posterior scalp proceeded to re-epithelialize through secondary intention healing six weeks after skin graft application. There were no instances of infection, bleeding, or hematoma.

## 4. Discussion

HS affects millions of patients with chronic symptoms and debilitating pain, significantly damaging their quality of life and psychosocial wellbeing [1,2]. In Hurley Stage I cases, pharmaceuticals are sufficient for symptom control. In more advanced disease (Hurley Stage II or III), wide surgical excision is imperative for cure and/or symptom reduction and control [6,26]. Following resection, rapid reconstruction is critical to prevent infection, reduce patient pain, and improve functional outcomes [27]. A prior prospective observational pilot study evaluating healing outcomes following tumescent-based excision performed by the same surgeon utilized a standard approach of skin grafting 3 weeks after excision. The present study, which utilized the same approach to surgical resection, demonstrated a reduced time to STSG application from 3 to 2 weeks, on average [28].

In the present study, an analysis of demographics within this patient population was similar to the distribution of HS in the broader population [29]. The average age of the patients included in the study was 32.6 ± 7.9 years. Within the larger population of HS patients, nearly half of the population was between the ages of 18 and 39 years, with a quarter of the population between 30 and 39 years [29]. While all eight patients in the present series self-identified as African American, this population is 2.5× more likely to develop HS than Caucasian individuals [29]. There was an even split between female and male patients enrolled, which varies slightly from the broader HS population [29]. Amongst the broader population, females are more likely to develop HS than males and represent nearly 3/4 of the population [29].

Advanced HS lesions are at high risk for disease recurrence due to chronic abscesses and infected sinus tracts. Moreover, they commonly occur in areas that are contaminated by coliform bacteria, such as the perineal and perianal regions. While diverting colostomies decrease the risk of frank fecal contamination, they do not eliminate the colonizing bacteria. Reconstruction of post-HS excision lesions have up to 25% surgical soft tissue infection rates [30]. SEFM may have unique anti-bacterial benefits in this patient population. Synthetic materials are resistant to enzymatic and bacterial degradation [21,22]. This allows for the SEFM to persist within volatile wound beds and achieve maximal healing results without concern for premature disintegration. In vitro studies of the SEFM conducted in accordance with United States Pharmacopeia (USP) <51> criteria for Antimicrobial Effectiveness Testing have demonstrated passing results against seven of the most commonly found microorganisms documented in non-healing wounds [22,31].

SEFM’s resistance to enzymatic degradation not only reduces the risk of infection that can be observed in biological materials but also supports organized wound healing responses. The SEFM is engineered to possess a resorption rate that matches that of tissue ingrowth [21], ensuring that it remains within the wound bed during the critical healing stages of inflammation and proliferation [32]. The varying pore and polymer sizes within the SEFM mimics native extracellular matrix, thus encouraging organized and structured healing responses [21]. A common post-operative complication in HS patients, especially in those who undergo radical or wide excision surgical approaches, is hyper granulation tissue formation. Despite the surgical approaches and subsequent post-excision management with both NPWT and SEFM, no instances of hyper granulation were observed, which could indicate that this combined approach may support organized healing responses.

Another challenge associated with the location of HS lesions is friction and shearing of STSGs. These lesions typically occur in intertriginous anatomy, such as axilla, groin, and perianal regions, making STSGs prone to failure [33]. STSG failure can be costly to both the patient and hospital systems alike, as it necessitates additional wound care and potentially re-operation [34]. Depending on the location of the wound, flap reconstruction may be considered. For axillary HS excisions, thoracodorsal artery perforator (TDAP) and propeller inner arm artery perforator (IAAP) flap procedures can be considered; however, complications remain quite high [34]. One retrospective comparative study of 13 patients conducted by Alabdulkareem et al. reported a total complication rate of 43% [34]. Of these patients, 21% experienced at least one flap-related complication, which were all within the IAAP group [34]. Flap procedures have longer operative times, with an average operating time of 201 min reported in Alabdulkareem et al. [34]. In addition to added expense, longer operative times are associated with an increased risk of complications such as infection, which is further increased in patients with high BMI [35,36,37].

In the present study, faster granulation of post-excision wound beds allowed for earlier STSG reconstruction. In the present study, one patient who experienced STSG loss proceeded to completely heal 6 weeks following graft application without additional complications or need for further procedures. Use of SEFM demonstrated robust granulation tissue formation, contributing to increased STSG incorporation and improved healing outcomes. The improved STSG incorporation and reduction in complication rates in the current study may suggest that use of the SEFM may improve grafting outcomes and limit the need for complex and expensive flap procedures, especially in patients who may not be able to tolerate these procedures or are prone to adverse healing outcomes.

The present study does have its limitations. While a total of 21 wounds are presented here, the small sample size of eight patients should be expanded in future studies. Additionally, there was no control group in this study, making head-to-head comparisons of outcomes challenging. SEFM was applied in conjunction with NPWT and an interpositional sheet of petrolatum gauze impregnated with 3% bismuth tribromophenate. Utilization of the gauze was intended to minimize adhesion of NPWT to the SEFM rather than augment wound healing or the chemistry of the wound environment. While bismuth has been shown to elicit antimicrobial effects, bismuth bound within the gauze has not been shown to leech and induce antimicrobial effects in surrounding tissue [38]. As a result, the use of petroleum gauze should be noted as a potential contributing factor to the overall wound healing outcomes observed. While use of dermal skin substitutes in conjunction with NPWT is well established in the literature [39,40,41], further investigation should be performed to elucidate the impact of each of these modalities on wound healing responses in this patient population. Future studies should be considered to address these concerns through the recruitment of a larger patient population and inclusion of one or more comparative treatment groups.

## 5. Conclusions

Excision of advanced HS lesions results in large soft tissue defects, many of which occur in topographically challenging regions that are prone to microbial contamination. In the present observational pilot study, a synthetic, electrospun polymer wound matrix was utilized to encourage vascularized tissue formation within post-HS excision wound beds for subsequent skin grafting. The electrospun, polymeric nature of the SEFM is engineered to resemble native human ECM, thus encouraging an organized cellular response for wound bed regeneration. This engineered design and composition offers a controlled rate of hydrolytic resorption which matches that of cellular ingrowth and neovascularization through the expanding porosity of the matrix during this process.

This pilot study of SEFM in conjunction with NPWT and bismuth-impregnated gauze successfully demonstrated rapid re-granulation of post-excision wound beds for skin grafting in an average of 2 weeks. Subsequent STSG reconstructed an average of 71% of the wound bed. No infections occurred throughout the entirety of the post-excisional period. These positive results indicate that this treatment approach supports robust granulation and STSG engraftment in these topographically challenging areas, demonstrating successful clinical translation of synthetic polymer materials.

## Figures and Tables

**Table 1:** Patient demographics and baseline wound characteristics. Age and body mass index are reported as mean ± SD for all patients included in the study. Post-excision wound surface is reported as the mean ± SD and range of all wounds treated in the study. Gender and race are reported as percentages of the study population. Wound locations are reported as the percentage of all wounds treated in the study. ^1^ SD = standard deviation.

Patient Demographic Information	
Gender (*n*, %)	
Male	4 (50%)
Female	4 (50%)
Patient age, years (mean ± SD ^1^)	
32.6 ± 7.9	
Race (*n*, %)	
African American	8 (100%)
Body Mass Index (mean ± SD ^1^)	
34.6 ± 3.8	
Post-excision wound surface area, cm^2^	
Mean ± SD ^1^	174.8 ± 139.6
Range	18–551
Wound location (*n*, %)	
Axillary	6 (28%)
Vulvar	4 (19%)
Inguinal	3 (14%)
Perianal	3 (14%)
Scrotal	1 (5%)
Perineal	1 (5%)
Abdomincal	1 (5%)
Mons	1 (5%)
Scalp	1 (5%)

**Table 2:** Primary and secondary outcomes. Time to split-thickness skin graft (STSG) is reported as time from hidradenitis suppurativa excision with synthetic electrospun fiber matrix application to skin graft application in days as mean ± standard deviation of all patients treated in the study. Successful STSG incorporation was reported as mean and standard deviation of all wounds grafted in the study, with instances of complete graft loss reported as 0% success. Complications are presented as a percent of total subjects. ^1^ STSG = split-thickness skin graft; ^2^ SD = standard deviation; ^3^ one instance of complete graft loss; ^4^ others included scrotal, perineal, abdominal, mons, and posterior scalp locations; ^5^ one instance of complete graft loss in the posterior scalp. The wound re-epithelialized through secondary intention healing 6 weeks after the skin grafting procedure.

Primary Outcome	
Time to STSG ^1^ (days) (mean ± SD ^2^)	
14 ± 3.2	
Secondary Outcomes	
Percent STSG ^1^ incoporation (mean ± SD ^2^)	
71% ± 28%	
Follow-up time (weeks)	
Mean ± SD ^2^	3.3 ± 3.0
Range	0.7–8.0
Percent STSG ^1^ incoporation by wound location (mean ± SD ^2^)	
Axillary	95% ± 11%
Vulvar	76% ± 12%
Inguinal	63% ± 6%
Perianal ^3^	40% ± 33%
Other ^4,5^	62% ± 33%
Complications (infection, bleeding, hematoma) (*n*, %)	
Complications	0 (0%)

## Data Availability

The original contributions presented in this study are included in the article. Further inquiries can be directed to the corresponding author.

## Abbreviations

The following abbreviations are used in this manuscript:

BMI	Body Mass Index
ECM	Extracellular Matrix
HIPPA	Health Insurance Portability and Accountability Act
HS	Hidradenitis Suppurativa
IRB	Institutional Review Board
NPWT	Negative Pressure Wound Therapy
OR	Operating Room
SD	Standard Deviation
SEFM	Synthetic Electrospun Fiber Matrix
STSG	Split-Thickness Skin Graft
USP	United States Pharmacopeia
